# Supplementary material for: Association between oral microbiome and seven types of cancers in East Asian population: a two-sample Mendelian randomization analysis
Source: Front Mol Biosci. 2023 Nov 21;10:1327893. doi: 10.3389/fmolb.2023.1327893 (PMC10702768; doi:10.3389/fmolb.2023.1327893)
Supplement: Supplementary file 1 [file DataSheet1.ZIP › Supplementary Materials/MR plots of saliva/saliva═╝/Breast cancer/pheno.3203_to_breast cancer_leave_one_out.pdf]

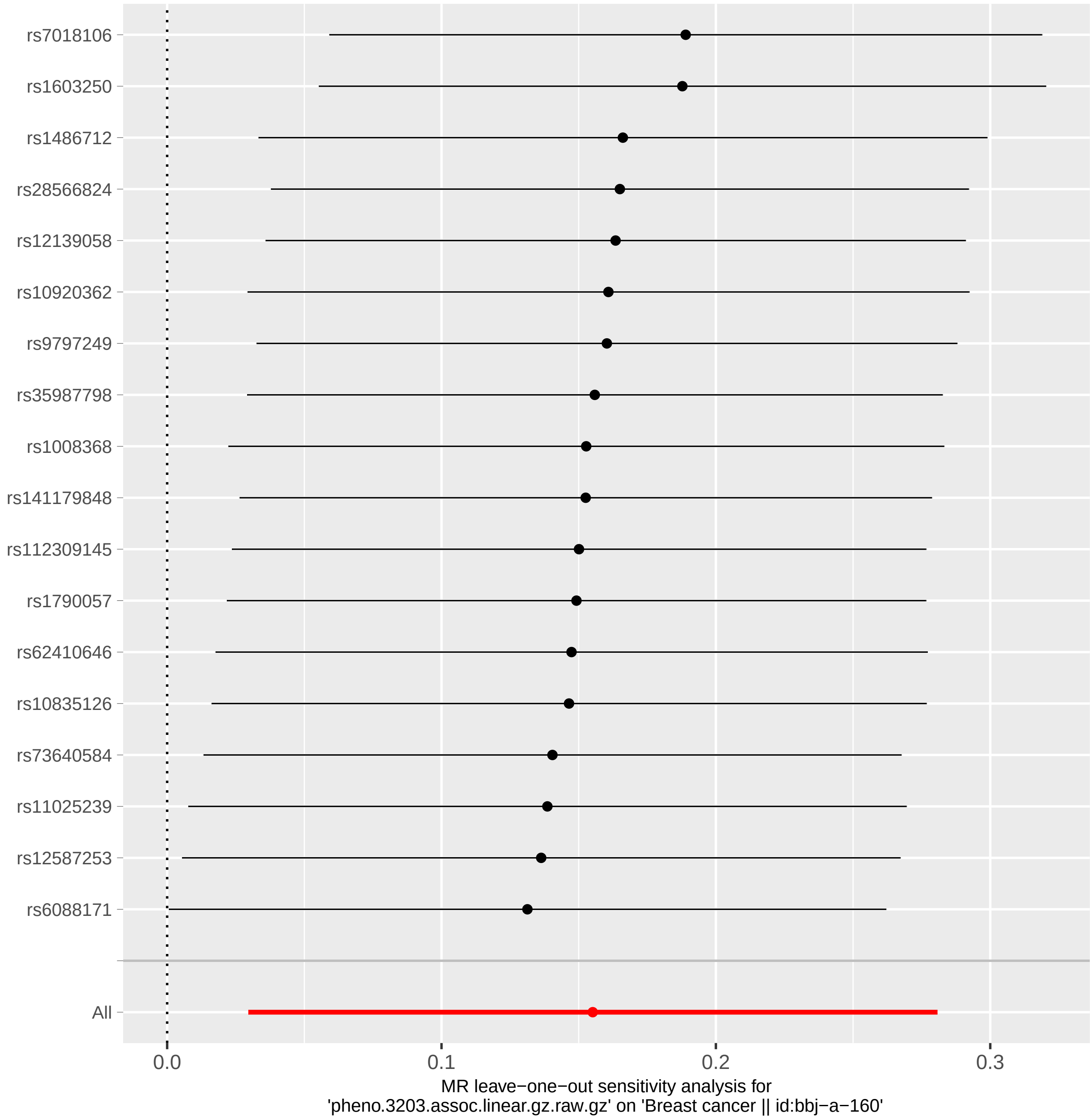
rs7018106
rs1603250
rs1486712
rs28566824
rs12139058
rs10920362
rs9797249
rs35987798
rs1008368
rs141179848
rs112309145
rs1790057
rs62410646
rs10835126
rs73640584
rs11025239
rs12587253
rs6088171
All
0.0
0.1
0.2
0.3
MR leave−one−out sensitivity analysis for
'pheno.3203.assoc.linear.gz.raw.gz' on 'Breast cancer || id:bbj−a−160'
